# Supplementary material for: Non-cascade random walks in solid-state high harmonic generation
Source: Nat Commun. 2026 Feb 18;17:2912. doi: 10.1038/s41467-026-69668-7 (PMC13031666; doi:10.1038/s41467-026-69668-7)
Supplement: Supplementary file 1 — Supplementary Information [file 41467_2026_69668_MOESM1_ESM.pdf]

Supplementary Information for Non-cascade Random Walks in Solid-state High Harmonic Generation

Zitan Zuo[1*], Yiwen Wang[1*], Shengzhe Pan[1§], Lulu Han[1], Yidan Xu[1], Dian Wu[1], Shicheng Jiang[1§], Jian Wu[1,2,3§]

[1]*State Key Laboratory of Precision Spectroscopy, East China Normal University, Shanghai 200241, China*

[2]*Collaborative Innovation Center of Extreme Optics, Shanxi University, Taiyuan, Shanxi 030006, China*

[3]*Chongqing Key Laboratory of Precision Optics, Chongqing Institute of East China Normal University, Chongqing 401121, China*

[*]*These authors contributed equally: Zitan Zuo, Yiwen Wang*

[§]*e-mail: szpan@lps.ecnu.edu.cn; scjiang@lps.ecnu.edu.cn; jwu@phy.ecnu.edu.cn*

Contents

Supplementary Note 1. Effects of relative phases among OAM modes

Supplementary Note 2. Random-walk behavior in multiphoton absorption process

Supplementary Note 3. Schematic of the experimental setup

Supplementary Note 4. Spin-orbit tomography for high-harmonic random walks

Supplementary Note 5. Programmability of high-harmonic random walks

Supplementary Note 1. Effects of relative phases among OAM modes

In the multiphoton absorption process of HHG, the phase coherence of the fundamental driving laser is inherently preserved and directly transferred to the emitted harmonics. Taking the fundamental beam as an example, when the rotation angle of QP is denoted as θ and the rotation angle of QWP is denoted as β. The constructed fundamental beam can be expressed as

$$|\psi_{\mathrm{H1}}\rangle = \frac{i}{2}e^{i\theta}|L,+1\rangle + \frac{1}{2}e^{-i(\theta+2\beta)}|L,-1\rangle + \frac{1}{2}e^{i(\theta-2\beta)}|R,+1\rangle + \frac{i}{2}e^{-i\theta}|R,-1\rangle.$$

The resulting second harmonic beam is given by

$$|\psi_{\mathrm{H2}}\rangle = \left(-e^{-2i\theta}|-2\rangle + 2ie^{-2i\beta}|0\rangle + e^{i(2\theta-4\beta)}|+2\rangle\right)|L\rangle$$
$$+\left(e^{-i(2\theta-4\beta)}|-2\rangle + 2ie^{2i\beta}|0\rangle - e^{2i\theta}|+2\rangle\right)|R\rangle.$$

Although the relative phase among OAM modes influences the spatial structure of the harmonic field—resulting in a rotation of the far-field intensity profile, as illustrated in Supplementary Fig. 1—it does not alter the probability distribution across OAM modes. This is because the observable probability in the OAM basis depends only on the squared amplitude of each mode and remains invariant under global or relative phase shifts. Therefore, while the relative phase can modulate the spatial arrangement of the output, it does not affect the core statistical outcomes of the random walk, which are the focus of this platform.

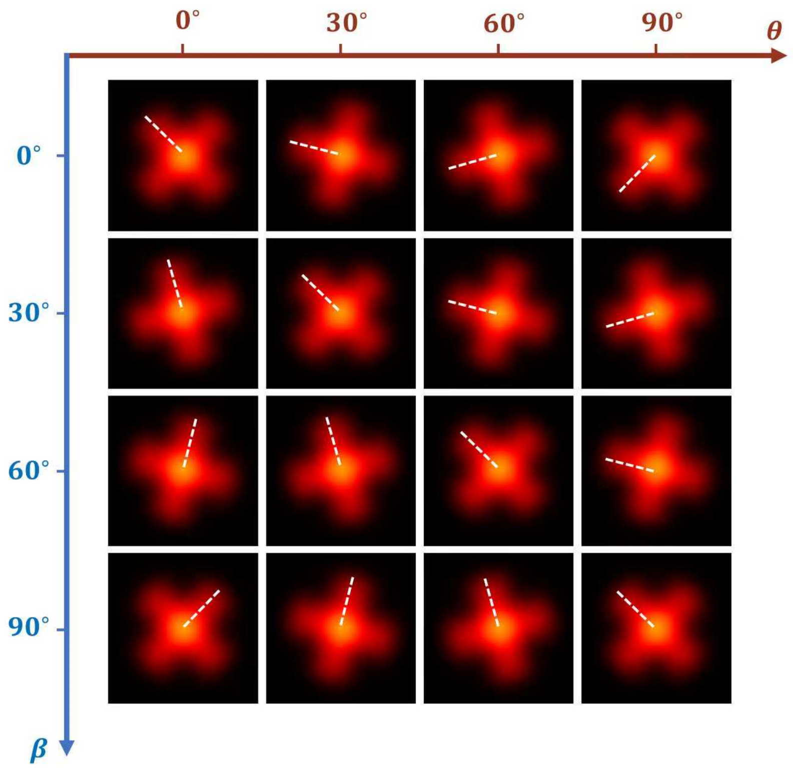

Supplementary Fig. 1. Influence of relative phase among OAM modes on the resulting second-harmonic intensity profiles. White dashed lines indicate the corresponding global rotation of the far-field patterns.

Supplementary Note 2. Random-walk behavior in multiphoton absorption process

The high-harmonic random walk realized in our work–arising from nonlinear electron dynamics in a crystal–represents a distinct class of non-cascade random walk rooted in solid-state HHG process. There exists a well-defined single-step operator that connects various photon absorption processes. Specifically, the HHG process can be interpreted in terms of cascaded *N*-photon absorption, governed by the following relation:

$$\beta^{(N)} = \left|\sum_{m_1+m_2+m_3+m_4=N} P^{(N)}_{m_1\sigma_1+m_2\sigma_2+m_3\sigma_3+m_4\sigma_4}\right|^2.$$

This can be recursively expressed as

$$P^{(N)}_{m_1\sigma_1+m_2\sigma_2+m_3\sigma_3+m_4\sigma_4} = C_1 P^{(N-1)}_{(m_1-1)\sigma_1+m_2\sigma_2+m_3\sigma_3+m_4\sigma_4} + C_2 P^{(N-1)}_{m_1\sigma_1+(m_2-1)\sigma_2+m_3\sigma_3+m_4\sigma_4} + C_3 P^{(N-1)}_{m_1\sigma_1+m_2\sigma_2+(m_3-1)\sigma_3+m_4\sigma_4} + C_4 P^{(N-1)}_{m_1\sigma_1+m_2\sigma_2+m_3\sigma_3+(m_4-1)\sigma_4},$$

where $P^{(N)}_{m_j\sigma_j}$ denotes the transition amplitude for absorbing m_j photons of angular momentum σ_j in generating the N-th harmonic, and C_j accounts for the number of quantum pathways leading to the transition. This formulation captures a cascaded absorption process, analogous to a step operator linking harmonic order $N-1$ to N. A similar interpretation has been established in the context of electron-light interactions, where sequential photon absorption gives rise to a random walk in the electron state space [1]. In our solid-state system, the situation is further enriched: beyond multiphoton absorption, the process also involves high-harmonic photon emission. This enables simultaneous, non-cascade emission across multiple harmonic orders, distinguishing our platform from conventional step-by-step quantum walks while preserving an underlying recursive structure.

Supplementary Note 3. Schematic of the experimental setup

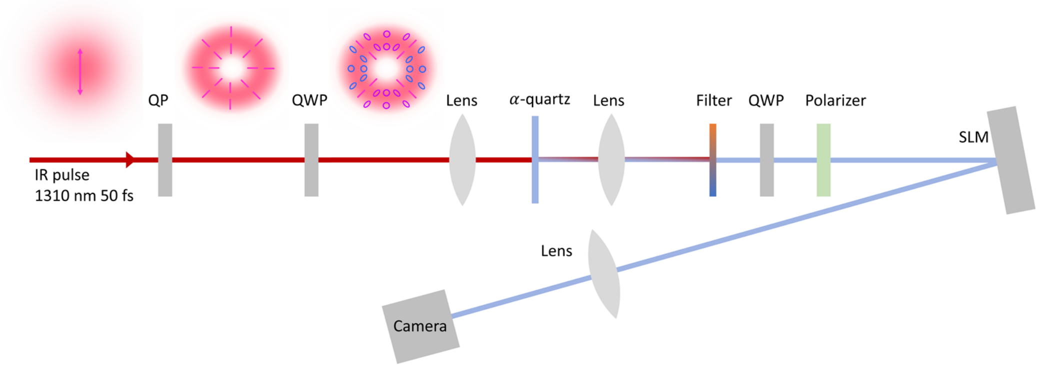

Supplementary Fig. 2. Schematic of the experimental setup. An incident linearly polarized Gaussian beam is converted into a radially polarized beam by a QP. The beam is then passed through a QWP, resulting in a complex vector beam whose polarization ellipticity varies continuously with the azimuthal angle, cycling from linear to circular and back to linear. This vector beam is focused by a lens onto a bulk α-quartz crystal to

generate high harmonics. Each harmonic beam can be selected using a band-pass filter. The polarization state of the selected harmonic is analyzed using a combination of a QWP and a polarizer. Meanwhile, its OAM components are characterized by diffraction from an SLM. The resulting beam profile is finally focused onto a CMOS camera for detection.

Supplementary Note 4. Spin-orbit tomography for high-harmonic random walks

Supplementary Fig. 3 presents the experimental spin-orbit tomography for the high-harmonic random walks. Although some discrepancies are observed, attributed to inhomogeneous harmonic emission patterns and the complexity of spin-orbit components, the measured OAM distributions show a good agreement with the simulated results, as summarized in Fig. 4 of the main text.

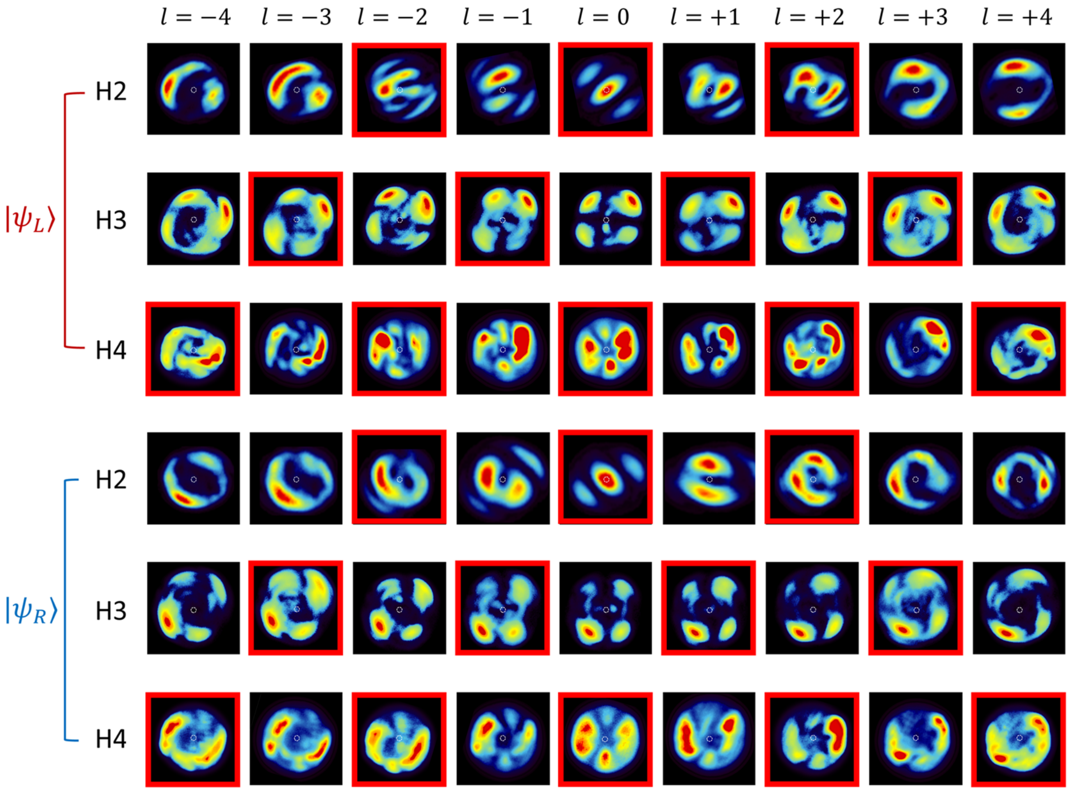

Supplementary Fig. 3. Experimental spin-orbit tomography for high-harmonic random walks. The top axis indicates the compensating topological charge $\Delta\ell$ applied via the spatial light modulator or spiral phase plates. Red squares highlight the configurations with distinct central spots, indicating the presence of a corresponding OAM state. Quantitative analysis of each OAM component is based on the integration of central zones in the images, marked by white dashed circles.

Supplementary Note 5. Programmability of high-harmonic random walks

The high-harmonic random walk platform can be effectively programmed using well-established optical-field-control techniques from ultrafast optics. Here, we propose a representative example based on a two-color scheme. The introduction of a second-harmonic (SH) laser field actively modulates the electron dynamics within the crystal and thereby enables access to different types of random walks. In addition to the pathways enabled by the fundamental laser field alone, the inclusion of an SH field with a state such as $|\psi_{\mathrm{SH}}\rangle = |L,+2\rangle + |R,-2\rangle$ opens additional photon absorption channels governed by

$$\Omega_n = \sum_j m_j\omega_j + \sum_k n_k(2\omega)_k.$$

Here, m_j and ω_j denote the number and frequency of absorbed j-th fundamental photon, n_k and $(2\omega)_k$ refer to the number and frequency of absorbed k-th SH photon.

To illustrate this programmability, we consider the generation of the third harmonic (H3) and assume equal contribution from two possible absorption pathways: $\omega+\omega+\omega$ and $\omega+2\omega$. Under these conditions, the resulting H3 signal can be described as

$$|\psi_{\mathrm{H3}}\rangle = (5i|-3\rangle - |-1\rangle + 3i|+1\rangle - 3|+3\rangle)|L\rangle$$
$$+(-3|-3\rangle + 3i|-1\rangle - |+1\rangle + 5i|+3\rangle)|R\rangle.$$

Supplementary Fig. 4a illustrates the resulting OAM distributions of H3 driven by a fundamental field (single-color in blue) and a fundamental field with an SH field (two-color in red), respectively. Notably, if the SH field contains only a left-handed circularly polarized component, namely, effectively implementing an unbalanced coin, the output

distribution becomes asymmetric, as yellow bars in Supplementary Fig. 4a. Similarly, the corresponding OAM distributions of the fourth harmonic, as the fourth-step random walk, are shown in Supplementary Fig. 4b.

This simulation confirms that through appropriate tailoring of the driving laser fields—such as frequency, polarization, or intensity—a high-harmonic random walk platform can be reconfigured to realize a range of distinct random walk behaviors, thereby demonstrating a meaningful degree of programmability.

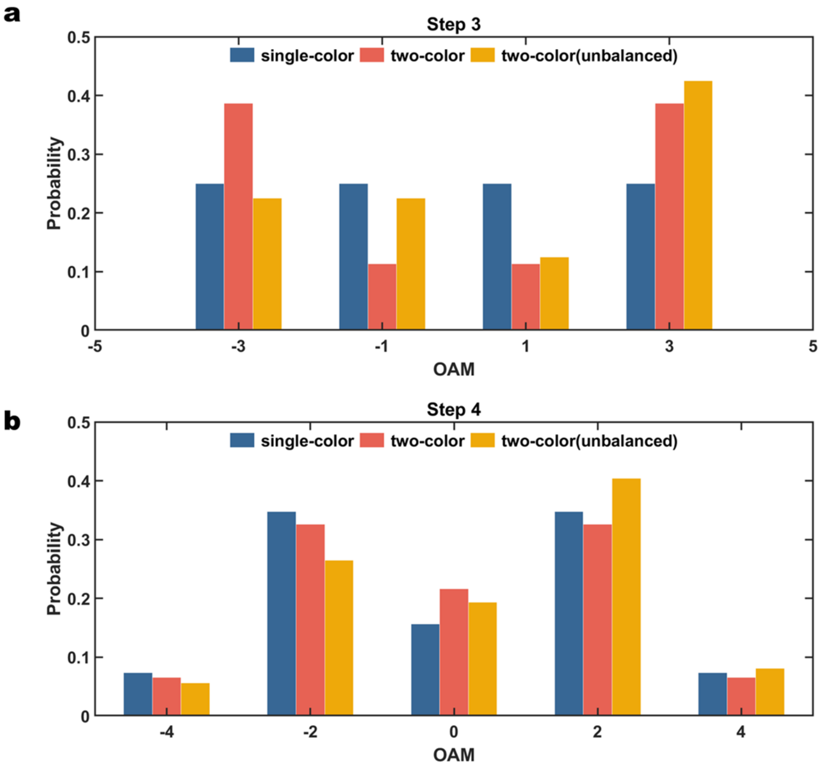

Supplementary Fig. 4. Programmable distribution of the high-harmonic random walk. (**a**) Probability distribution of the third-step high-harmonic random walks driven by a single-color field, a balanced two-color field, and an unbalanced two-color field, respectively. (**b**) Probability distribution of the fourth-step high-harmonic random walks driven by a single-color field, a balanced two-color field, and an unbalanced two-color field, respectively.

Supplementary References:

[1] Dahan, R. et al. Imprinting the quantum statistics of photons on free electrons. *Science* **373**, eabj7128 (2021).
